# Boehmite Nanofillers in Epoxy Oligosiloxane Resins: Influencing the Curing Process by Complex Physical and Chemical Interactions

**DOI:** 10.3390/ma12091513

**Published:** 2019-05-09

**Authors:** Ievgeniia Topolniak, Vasile-Dan Hodoroaba, Dietmar Pfeifer, Ulrike Braun, Heinz Sturm

**Affiliations:** 1 Federal Institute for Material Research and Testing (BAM), 12205 Berlin, Germany; Dan.Hodoroaba@bam.de (V.-D.H.); Dietmar.Pfeifer-Berlin@t-online.de (D.P.); ulrike.braun@bam.de (U.B.); 2 Institute Machine Tools and Factory Management, Technical University Berlin, 10587 Berlin, Germany

**Keywords:** boehmite, nanocomposite, cationic photocuring, cycloaliphatic epoxy oligosiloxane, epoxy conversion degree, real-time infrared spectroscopy

## Abstract

In this work, a novel boehmite (BA)-embedded organic/inorganic nanocomposite coating based on cycloaliphatic epoxy oligosiloxane (CEOS) resin was fabricated applying UV-induced cationic polymerization. The main changes of the material behavior caused by the nanofiller were investigated with regard to its photocuring kinetics, thermal stability, and glass transition. The role of the particle surface was of particular interest, thus, unmodified nanoparticles (HP14) and particles modified with p-toluenesulfonic acid (OS1) were incorporated into a CEOS matrix in the concentration range of 1–10 wt.%. Resulting nanocomposites exhibited improved thermal properties, with the glass transition temperature (T_g_) being shifted from 30 °C for unfilled CEOS to 54 °C (2 wt.% HP14) and 73 °C (2 wt.% OS1) for filled CEOS. Additionally, TGA analysis showed increased thermal stability of samples filled with nanoparticles. An attractive interaction between boehmite and CEOS matrix influenced the curing. Real-time infrared spectroscopy (RT-IR) experiments demonstrated that the epoxide conversion rate of nanocomposites was slightly increased compared to neat resin. The beneficial role of the BA can be explained by the participation of hydroxyl groups at the particle surface in photopolymerization processes and by the complementary contribution of p-toluenesulfonic acid surface modifier and water molecules introduced into the system with nanoparticles.

## 1. Introduction

Organic–inorganic nanostructured materials such as nanocomposites and hybrid materials have attracted growing interest during the last decade, particularly due to their extraordinary properties resulting from the structure of alternating organic and inorganic components. A relevant difference between hybrids and nanocomposites is the fact that in the former ones the inorganic phase is formed in situ, for instance by a sol-gel process, while nanocomposites are usually produced by dispersing inorganic particles with at least one dimension less than 100 nm in a polymer matrix. 

The use of inorganic filler in the nanoscale range is a well-known approach to enhance the specific properties of polymers, e.g., thermal, electrical, optical, mechanical or barrier properties [1,2]. The effect of a filler on the resultant nanocomposite depends on different factors including the particle shape, size, loading, surface properties, agglomeration, and dispersion in the matrix. Therefore, it is quite often hard to predict an overall change in polymeric material upon nanofiller inclusion. 

Boehmite alumina (BA) shows promising results as a nanofiller. It was shown that incorporation of these inorganic nanoparticles into polymers resulted in modification of their characteristics, in particular, surface hardness [3], fire retardancy [4], electrical [5], thermal [5], mechanical [6], and barrier properties [7]. Boehmite is an aluminum oxyhydroxide containing the crystalline part known as γ-AlO(OH) along with a pseudo-boehmite part which includes some water molecules [8]. The surface of BA can be easily modified enhancing the dispersion of the particles in various polymers. In combination with ease of particle customization, this sparked a significant amount of research performed on the integration of BA filler in thermoplastics as well as in thermosets [7]. 

Another path to achieve superior material properties is by implementing hybrid materials where the advantageous properties of the polymeric materials and the inorganic structures are combined. In these materials, organic and inorganic segments are covalently bonded and interpenetrate each other on a few nanometers to a few micrometer ranges [9]. Among this class of materials, siloxane-based hybrids have several advantages for material design. They are easy to synthesize or to modify with a broad variety of commercially available precursors, easy to process, are non-toxic materials exhibiting good transparency and superior mechanical properties which can be tuned between those of glasses and those of polymers [10]. 

Applying the photocuring technology to produce hybrid coating offers numerous benefits covering a considerable sector of industrial production. One of the promising areas in the photocuring industry is cationic photopolymerization. In contrast to free-radical polymerization, cationic photopolymerization is not inhibited by oxygen and exhibits a low degree of shrinkage. Furthermore, a very long lifetime of active species allows dark-curing and thermal post-curing after UV light initiation [11,12,13]. However, one of the major limitations of this process is low reactivity of such epoxies, especially compared with acrylate-based hybrids. Cycloaliphatic epoxy formulations are the most reactive, and therefore, extensively applied in the production of cationic UV-cured coatings [14]. Nevertheless, extremely high crosslinking density of this resin leads to the poor toughness of cured films. To overcome this drawback and to achieve superior performance of cycloaliphatic epoxy resins, the introduction of oligomers and pre-polymers into the resin network can be applied [15,16]. 

Ultraviolet-curable cycloaliphatic epoxy oligosiloxane (CEOS) resin synthesized by simple non-hydrolytic sol-gel reaction [17] was shown to be a material with superior properties. On one side, implemented cycloaliphatic epoxy groups make it easy to process thin films at ambient temperatures in a short time-frame. At the same time, CEOS displays good thermal stability, high refractive index, low permeability, and was reported to be a good candidate for the encapsulation of flexible organic electronics [18]. Nevertheless, nowadays, multifunctionality in a material formulation is essential. For instance, coatings do not only play a protection role, but also deliver supplementary functions including, but not limited to high adhesion and stability, chemical and scratch resistance, and high durability [19]. Therefore, further reinforcement of the CEOS properties holds a great interest for advanced material applications. It was shown that embedding of silica nanoparticles leads to enhanced barrier properties of CEOS films [18]. However, the nature of the enhancement of its properties with different nanofillers has not been fully established. The boehmite nanofillers carry potential due to the versatility of particle morphology and surface properties. As a result, incorporation of well-tailored BA into the CEOS matrix could yield a flexible, transparent resin layer with advanced functional characteristics, which could be advantageous in applications where short-time processing at ambient temperatures is required. This reflects the potential of this material in fields like microfluidics, organic electronics or 3D micro-structuring. 

The current study aims at investigating the preparation and characterization of a novel UV-cured boehmite-embedded cycloaliphatic epoxy oligosiloxane resin films. To determine the role of particle–matrix interphase in nanocomposite properties unmodified and surface modified (with p-toluenesulfonic acid) boehmite nanoparticles were applied. The effect of surface modification was verified by scanning electron microscopy (SEM) operated in transmission mode where the particles’ distribution was evaluated. The curing behavior was studied by means of real-time infrared spectroscopy (RT-IR) and the thermal behavior of the cured networks were analyzed with differential scanning calorimetry (DSC) and thermogravimetric analysis (TGA). The suggested analytical approach allowed to correlate the evolution of curing degree, thermal events, and presence/absence of filler interaction with polymeric network with respect to BA loading, filler distribution, and presence of surface modifier. Consequently, possible hypotheses of boehmite influence on CEOS behavior were discussed. 

## 2. Materials and Methods

### 2.1. Materials

Commercially available boehmite alumina nanoparticles (BA) without surface modifier (DISPERAL^®^, HP14) and modified with p-toluenesulfonic acid (DISPERAL^®^, OS1) were supplied by Sasol, Hamburg, Germany. Selected particle parameters are presented in Table 1. The chemical structures can be found elsewhere [20] (Supplementary Materials Figure S1). 

Cycloaliphatic epoxy oligosiloxane (CEOS) was synthesized via non-hydrolytic sol-gel reaction and used as a UV curable matrix. The synthesis procedure is described below. Tetrahydropyran (THF, 99.9% for HPLC, Chemsolute, Berlin, Germany) was used as a solvent for nanocomposite preparation without further purification. 

### 2.2. Synthesis of Cycloaliphatic Epoxy Oligosiloxane (CEOS)

**Scheme 1:**
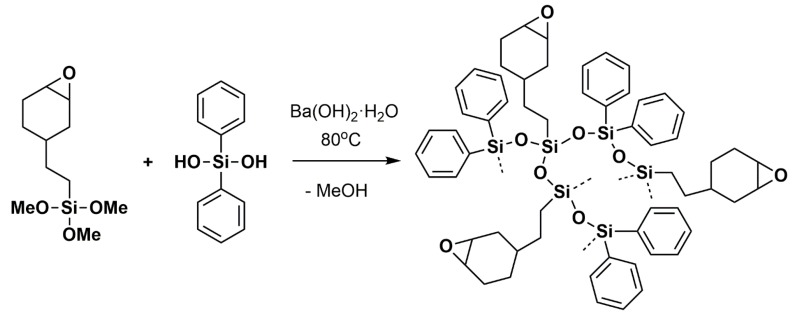
Synthesis of cycloaliphatic epoxy oligosiloxane (CEOS) by a sol-gel condensation.

The CEOS was synthesized by a condensation reaction between 2-(3,4-epoxycyclohexyl) ethyltrimethoxysilane (ECTS, 99.8%, Sigma–Aldrich, St. Louis, MO, USA) and diphenylsilanediol (DPSD, 98%, Alfa Aesar, Tewksbury, MA, USA) in the presence of barium hydroxide monohydrate (Honeywell, Düsseldorf, Germany) as a catalyst. The schematic representation of reaction is shown in Scheme 1. For the sake of achieving high condensation yield, different ECTS:DPSD molar ratios were investigated. The amount of added catalyst was 0.2 mol % equivalents of ECTS in the mixture. The DPSD was continuously added to the ECTS in the flask under N_2_ atmosphere while stirring the solution with a magnetic stirrer for 2 h at 80 °C and additional 2 h at room temperature to complete the reaction. After the reaction ended, the flask was vacuum heated to remove methanol formed as a by-product during the condensation. Ba(OH)_2_·H_2_O was removed using 0.45-μm pore-sized PTFE filter. The detailed synthesis procedure is also described in detail elsewhere [17]. As a result, a colorless, viscous solution of CEOS was obtained. 

Triarylsulfonium hexafluorophosphate salt (50 % in propylene carbonate, Sigma-Aldrich, Steinheim, Germany) was added to CEOS as a cationic photoinitiator (PI) to enable further UV photocuring reaction. The concentration of PI was fixed at 2 wt.% with regard to CEOS amount. 

### 2.3. Nanocomposite Preparation and Film Curing

Boehmite suspension in THF was placed in an ultrasonic bath for 30 min and then treated by an ultrasonic homogenizer (Sonopuls HP4200, Bandelin, Berlin, Germany) for 15 min immediately preceding the mixing with CEOS/PI solution. To tailor the final viscosity, THF was added to CEOS prior to mixing with the nanoparticles. After sonication, aliquots of BA suspensions were added to CEOS solution so that formulations with filler loadings between 1 and 10 wt.% in the final cured solid-state hybrid were achieved. The nanocomposite solutions were sonicated for 15 min with ultrasonic homogenizer to ensure homogeneous distribution of the particles. Next, depending on the further characterization method, films with different thickness/area size were obtained by either spin-coating or bar-coating techniques. Due to the different sample sizes required, two different UV light sources were used for CEOS curing. A set of Hg–Xe lamp Hamamatsu LC8 (Hamamatsu, Iwata, Japan) with an optical waveguide and A9616-05 bandpass filter (Schott, Mainz, Germany) was used for irradiating small sample areas (UV dose of 0.12 mJ/cm^2^/sec) as it was needed for the monitoring of curing kinetic or to prepare the samples for the BA distribution investigations. To obtain large-area samples, the Vacuum-UV-Exposure-Box-1 (Gie-Tec, Eiterfeld, Germany) equipped with low-pressure Hg-vapor gas-discharge lamps was used to expose CEOS and nanocomposite films (UV dose of 2.2 mJ/cm^2^/sec for 15 min). These samples were subject of the thermal analysis. 

### 2.4. Characterization

The condensation reaction between DPSD and ECTS was verified by ^29^Si and ^13^C nuclear magnetic resonance (NMR) where the spectra of CEOS solution in chloroform-d were recorded with FT 600 MHz (Bruker, Karlsruhe, Germany) spectrometer. Tetramethylsilane was used as an internal reference. 

Differential scanning calorimetry (DSC) analysis was performed on a DSC 7020 (Hitachi, Tokyo, Japan) instrument to determine the glass transition temperature (T_g_) of CEOS and various CEOS/BA-cured films. The films of different nanocomposite formulations were obtained by exposing bar-coated films to UV light for 15 min using the exposure box. The samples of the weight around 2.5 mg were heated in a sealed Al pan at the ramp of 10 °C /min under N_2_ atmosphere. The scanning range was between 50 °C and 250 °C. In a DSC scan, T_g_ was detected as a temperature at a half-height of heat flow step. 

Thermogravimetric analysis (TGA) was carried out with 10 mg of samples using a TGA/SDTA 851 (Mettler Toledo, Greifensee, Switzerland) unit at the heating rate of 10 °C/min under N_2_ atmosphere. The same sample batch as that analyzed by DSC was investigated by TGA. 

Boehmite nanoparticles and their distribution in the CEOS films were investigated by SEM using a Zeiss Supra 40 microscope (Carl Zeiss, Oberkochen, Germany) equipped with a high-resolution cathode of type Schottky–Feld emitter and conventional secondary electron (SE) and In-Lens secondary electron (In-Lens) detectors. For better observation of the particles within the sample volume, SEM was operated in the transmission mode, i.e., the so-called T-SEM, whereby a dedicated sample holder was used [22]. Hence, the superior material contrast of the T-SEM operation mode could be exploited for the observation of the particles in the polymeric matrix. Boehmite suspension in THF was deposited on the substrate and observed after the solvent was evaporated. The samples for T-SEM were prepared by curing the spin-coated thin films of CEOS and its nanocomposites on the KBr-based substrate followed by dissolving the substrate in Millipore purified water. Resultant free-standing CEOS-based films were deposited on transmission electron microscopy (Plano, Wetzlar, Germany) grid with the support carbon film. 

Photocuring kinetic was followed by RT-IR spectroscopy conducted in transmission mode using a Nicolet 8700 FTIR spectrometer (Thermo Electron Corporation, Madison, WI, USA). The samples were prepared by spin-coating on the KBr substrates from CEOS solutions in THF and its mixtures with BA nanoparticles. As a result, films with thicknesses ranging from 10–20 μm were obtained. The samples were placed perpendicularly to the IR beam direction. The mid-IR spectra were collected in the 650–4000 cm^−1^ wavelength range at a resolution of 4 cm^−1^ with the acquisition time of 5.5 s. The total time of UV exposure was 30 min. Additionally, the IR spectra of the samples were measured after 24 h of keeping them in the darkness to evaluate the impact of post-curing processes. To reduce variability, all RT-IR experiments were performed on the same day and under the same conditions. Experiments were performed at least in triplicate for each formulation. 

The conversion of epoxy groups α_ep_ was calculated by following equation:

(1) αep=1−[(AepoxyAaromatic)t(AepoxyAaromatic)t=0]×100%

where *A_epoxy_,* the peak area of absorption band of epoxy group at 885 cm^−1^, is normalized by the peak area of Si–Ph group located at 1430 cm^−1^ [23], abbreviated as *A_aromatic_*. The peak of Si–Ph was chosen as a reference due to the assumption that the number of Si–Ph bonds remains constant during the polymerization. 

## 3. Results and Discussion

### 3.1. Characterization of Cycloaliphatic Epoxy Oligosiloxane (CEOS)

**Figure 1:**
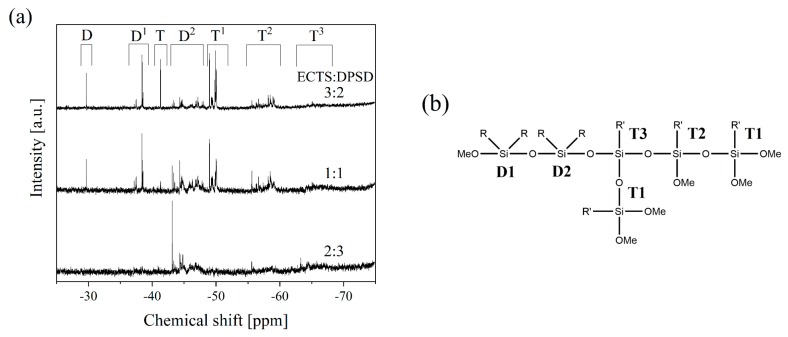
(**a**) ^29^Si NMR spectra of CEOS synthesized at different ECTS:DPSD molar ratios; (**b**) Representation of Si atoms with different bond states (R: phenyl; R′: 2(3,4-epoxycyclohexyl)ethyl).

The molecular structure of synthesized CEOS was characterized by ^13^C NMR, ^29^Si NMR, and FTIR spectroscopy. Figure 1 displays ^29^Si NMR spectra of CEOS with different ECTS:DPSD adduct ratios. From the CEOS structure, Si atoms can be assigned to DPSD and ECTS parts, denoted as D^n^ and T^n^, respectively (Figure 1b). The superscript indicates the number of siloxane bonds of Si atom. 

As one can see in Figure 1b, D and T peaks located at −29.7 ppm and −41.2 ppm, respectively, represent product of DPSD dimerization and unreacted ECTS molecules [17]. Their absence in CEOS with the highest DPSD amount signify that the reaction had successfully proceeded, enabling the formation of epoxy-contained oligosiloxane. Furthermore, the increase of DPSD concentration leads to more condensed structure as indicated by higher intensity of D^2^, T^2^, and T^3^ peaks compared to peaks of Si with only one siloxane bond formed (D^1^, T^1^). This observation is in good agreement with previous investigations of CEOS synthesis [17,24]. Due to higher reaction yield, ECTS:DPSD molar ratio of adducts was chosen as 2:3 for further nanocomposite processing. It is important to mention that epoxy groups remained stable during condensation reaction as no opening of oxirane ring was observed at ^13^C NMR spectra (Supplementary Materials Figure S2), where all CH–O signals appear at ~52 ppm (oxirane site) but not at 55–70 ppm (opened oxirane ring). 

**Figure 2:**
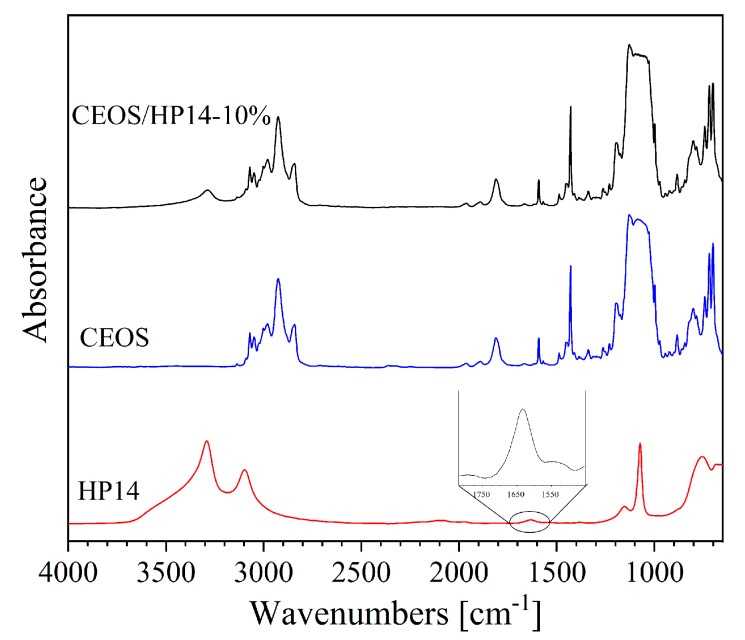
FTIR spectra of neat CEOS, unmodified BA nanoparticles HP14, and CEOS/HP14-10% before curing.

The FTIR spectrum of neat CEOS before curing was recorded and compared with unmodified boehmite particles (HP14) and resultant CEOS/HP14 mixture with 10 wt.% nanoparticle loading (Figure 2). Characteristic IR bands of CEOS and BA are summarized in Supplementary Materials Table S1. 

As expected from the synthesis route, FTIR spectrum of CEOS thin film did not exhibit any Si–OH peak in the range between 3700 and 3200 cm^−1^ [23]. Neither hydroxyl groups were observed, which confirms that the oxirane ring had not been opened during the synthesis. Newly formed Si–O–Si bonds produced an absorption in the range of 1040–1090 cm^−1^. Nevertheless, it should be noted that the peak observed at 2840 cm^−1^ represents residual Si–OMe groups which can be also confirmed by the presence of a T^2^ structure reported by ^29^Si NMR (Figure 1). The vibration of the epoxy ring, detected at 885 cm^−1^, was not affected by BA addition. 

Typical spectrum of boehmite, as shown in Figure 2, displays a sharp peak at 1064 cm^−1^ and shoulder bands at 3100 and 3290 cm^−1^, which were assigned to bending and stretching vibrations of OH groups in BA phase, respectively. A peak at 1640 cm^−1^ was typical for OH vibration in water molecules indicating the presence of physically adsorbed water in BA powder. The presence of water can be corroborated by a shoulder at around 3500 cm^−1^. The spectrum of organophilic boehmite particles OS1 modified with p-toluenesulfonic acid (PTSA) exhibits the similar bands as those of unmodified HP14 with additional peaks in the range of 1010–1170 cm^−1^ associated with PTSA [25] (Supplementary Materials Figure S3). It was observed that the FTIR spectrum of nanocomposite displayed the combination of both CEOS and BA. Despite an overlap of most vibration bands of the two components, no formation of new peaks or peak shifts signifying chemical reaction was observed after mixing CEOS and BA. 

### 3.2. Thermal Analysis of Cured CEOS and CEOS/BA Nanocomposites

**Figure 3:**
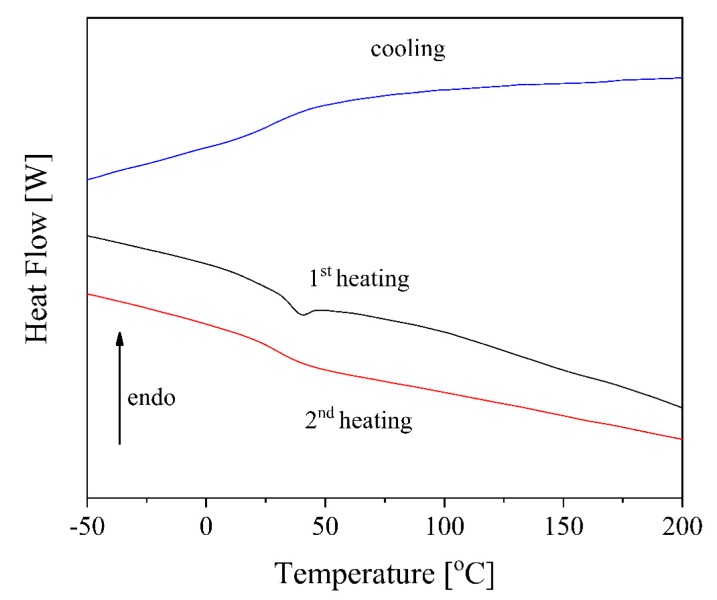
DSC thermogram of neat CEOS hybrid.

Differential scanning calorimetry (DSC) was applied to investigate the thermal characteristics of neat CEOS and CEOS/BA nanocomposites. Figure 3 displays the thermal behavior of CEOS at first and second heating and cooling scans. The glass transition temperature (T_g_), appearing as an endothermic step change, can be detected at 22 °C and 30 °C for the first and second heating, respectively. At the same time, an endothermic relaxation peak was observed during glass transition at the first heating cycle. This relaxation corresponds to a rearrangement in the molecules to release the stress frozen at temperatures below T_g_ [26]. Therefore, it is no longer observed on cooling or second heating scans. In this study, only the second heating scan was considered for further analysis. 

It was reported that an increase in T_g_ was detected with an increase of the curing degree of thermosets [27]. Based on that, the glass transition temperature can be considered highly dependent on the curing process. In contrast to gelation (liquid-to-rubber), vitrification (rubber-to-glass) is a reversible phenomenon and can be eliminated by increasing the curing temperature above T_g_. However, photocuring, which is usually carried out at ambient temperatures, suffers from early vitrification resulting in hindered monomer crosslinking and low overall curing degree. This effect can be recognized in this experiment. As the CEOS resin was photocured at room temperature, the low value of detected T_g_ represents the described limitation due to an early approach to a glassy state. 

Analyzing the second heating scan of neat CEOS and its nanocomposites (Supplementary Materials Figures S4–S7), the results of the thermogram analysis have been summarized in Table 2. One can see that the glass transition shifts to higher temperatures with particle addition for all nanocomposite formulations. It indicates the hindrance effect of BA on segmental motions of CEOS network, what usually results from the attractive nature of nanoparticle–polymer interactions. However, thermal behavior of nanocomposites differs depending on the used filler. Higher enhancement of T_g_ was observed for CEOS/OS1 formulations, where boehmite surface was modified with p-toluenesulfonic acid. At the same time, in contrast to CEOS/HP14, the glass transition step ΔC_p_ of CEOS/OS1 films becomes less pronounced with an increase of the filler amount as can be observed from thermograms (Supplementary Materials Figures S4–S7) and ΔC_p_ values in Table 2. The highest T_g_ rise was detected for CEOS/HP14-2% and CEOS/OS1-2% reaching values of 54 °C and 73 °C, compared to neat polymer, respectively. 

**Figure 4:**
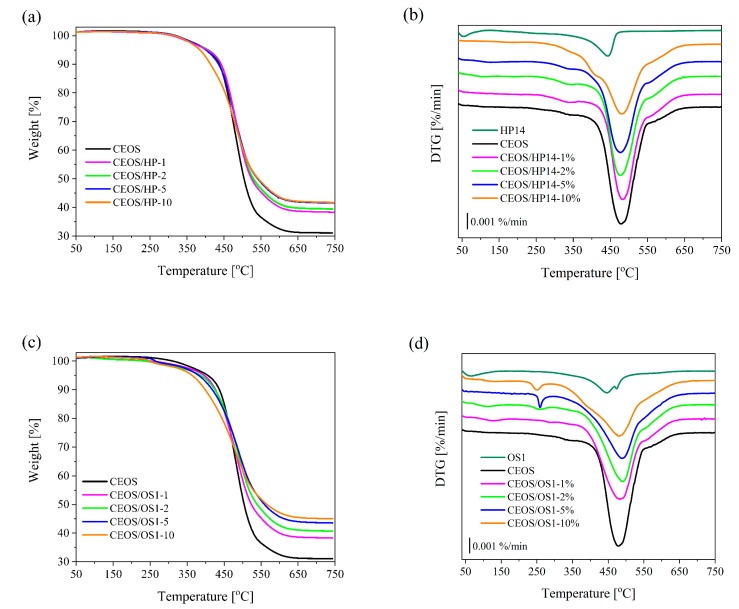
(**a**,**c**) TG curves of CEOS resin with different loadings of (**a**) unmodified boehmite, HP14 and (**c**) organically-modified boehmite, OS1; (**b**,**d**) differential thermal gravimetric (DTG) curves of BA powder, neat CEOS and CEOS/BA nanocomposites with 1–10 wt.% of BA contents: (**b**) with unmodified boehmite, HP14, and (**d**) with organically-modified boehmite, OS1.

To complement thermal analysis, TGA was performed to evaluate the influence of BA on CEOS stability at high temperatures. Figure 4 depicts the normalized TG curves (a,c) and their derivatives (b,d) for the neat CEOS, the BA nanoparticles and different nanocomposite formulations. For comparison, the detailed description of TG behavior of boehmite nanoparticles is given in Supplementary Information (Supplementary Materials Figure S8). First, one can mention the high thermal stability of the CEOS network. The 5% weight loss temperature of CEOS was detected at 410 °C what is similar to the value reported previously [17]. As observed in Figure 4a, the temperature range of decomposition remains almost the same for CEOS/HP14 with 1–5 wt.% filler concentration, but starts to decrease for film with 10 wt.% of HP14 loading. 

Differential thermogravimetric (DTG) curves (Figure 4b,d) show that the main decomposition step of CEOS resin starts at 420 °C with the maximum rate located at 480 °C. However, the dihydroxylation of BA particles (Figure 4b,d), begins already at 380–390 °C. Boehmite presence leads to the broadening of CEOS degradation peak due to the superposition of both hybrid and filler peaks. However, the addition of boehmite filler does not postpone the main decomposition event. 

**Figure 5:**
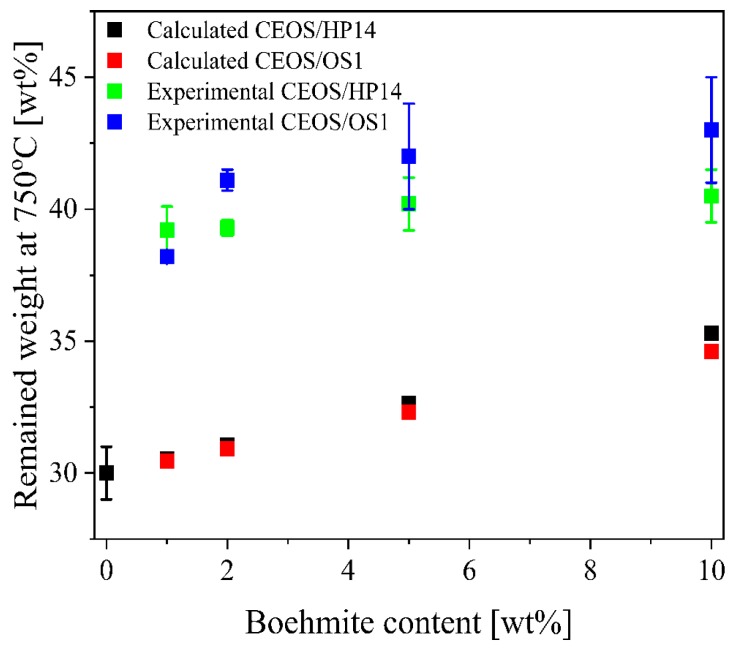
Comparison between expected and measured values of residue weight of CEOs and CEOS/BA nanocomposites as a function of filler content.

On the other hand, the reinforcement of overall temperature resistance of the films with boehmite addition can be detected. Figure 5 displays the evolution of residual weight detected for analyzed formulations at 750 °C, after the main decomposition associated with the organic segment in CEOS has occurred. Comparing these values with those calculated from superposition principle, the experimental data are considerably higher. This could indicate the formation of highly stable species as a result of attractive filler-resin interactions. 

### 3.3. Boehmite Distribution

**Figure 6:**
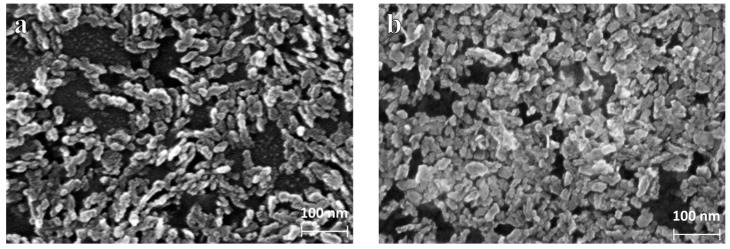
10 kV SEM micrographs of boehmite nanoparticles obtained with In-Lens detector: (**a**) HP14 deposited on Si wafer; (**b**) OS1 deposited on carbon film.

The particle morphology and more importantly, their distribution within the epoxy matrix have a significant impact on the properties of resultant nanocomposite materials. Strong particle agglomeration and aggregation can lead to inhomogeneity of the material properties and indicates poor interaction between organic and inorganic components. To ensure the sufficient boehmite distribution in the hybrid network, nanocomposite films were verified by means of scanning electron microscopy operated in transmission mode (T-SEM). For the sake of highly sensitive surface analysis, e.g., particles morphology, SEM with surface-sensitive In-Lens detector was applied. Comparing boehmite particles without and with surface modifier (Figure 6), one can see that unmodified HP14 and PTSA-modified OS1 exhibit similar morphology. It is known that γ-AlO(OH) has an orthorhombic unit cell [28,29]. Considering the size of BA crystallites reported in Table 1 (14 nm and 10 nm for HP14 and OS1, respectively), one assumes that the observed 10–50 nm long particles are mostly aggregates made up of several boehmite single crystallites. Meanwhile, the widths of the particles differ, being in the ranges of 10–15 nm and 10–20 nm for HP14 and OS1, respectively. This indicates mostly monocrystallite width. Increased width of OS1 particles can be a result of surface treatment with PTSA. 

**Figure 7:**
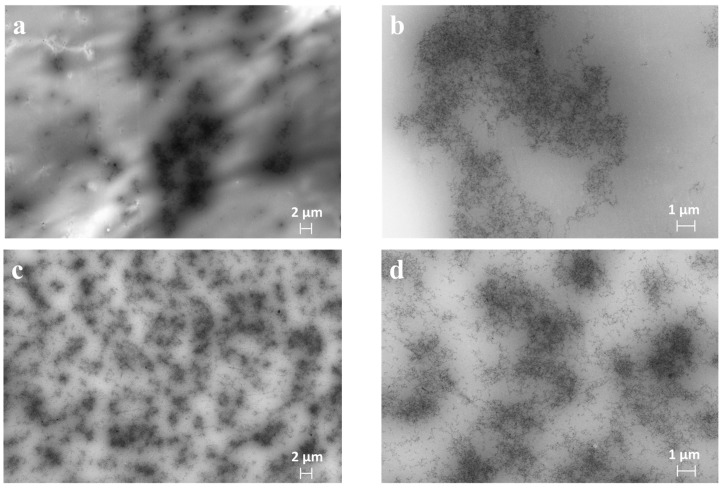
20 kV T-SEM micrographs of (**a**,**b**) CEOS/HP14 and (**c**,**d**) CEOS/OS1 nanocomposite films with 5 wt.% of boehmite content.

While HP14 particles are compatible with polar environment, organophilic OS1 are meant to be used in medium polar matrices. As expected, this has caused the difference in the filler distribution in CEOS films. Figure 7 shows that HP14 in general forms agglomerates of considerably larger size than those of OS1, as a result of different compatibility between the resin and the particles. Nevertheless, surface modification of boehmite does not have any noticeable impact on the particle dispersion within agglomerate as it appears to be alike for both HP14 and OS1 (Figure 7b,d). 

**Figure 8:**
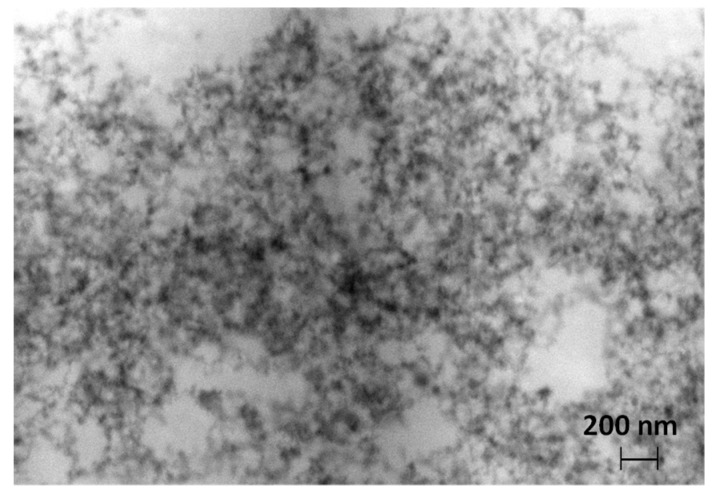
20 kV SEM micrographs of boehmite agglomerates in the CEOS/OS1 film obtained in the transmission mode.

It is worth noting that boehmite agglomerates appear as clusters of network-like-connected particles (Figure 8). This agglomerate structure causes the formation of a large-area matrix–boehmite interface what is a precondition for enhanced resin-filler interaction. 

One can suspect that better distribution of organically-modified OS1 clusters in the CEOS matrix leads to a higher degree and better homogeneity of structuration in nanocomposites as well as to improved interaction with the polymer compared to the unmodified nanoparticles. This is supported by enhanced effect on T_g_ shift for CEOS/OS1 formulations, which indicates the hindrance of the motions of polymer chain and a decrease of free-volume by well-dispersed OS1 particles. 

### 3.4. UV Curing Kinetics

The properties of thermosets strongly depend on the curing parameters such as curing procedure, degree and depth of curing, post-curing treatments, etc. To better understand the reasons for the observed thermal behavior of nanocomposites, the photocuring of CEOS and all CEOS/BA formulations were investigated in situ by means of RT-IR spectroscopy. The thickness of all studied formulations was kept similar (10–15 μm) and measured after light exposure when the solid film was obtained. In this range of coating thicknesses and since the high-energy UV source was applied, the differences in penetration depth of the UV light between different samples can be neglected. 

**Figure 9:**
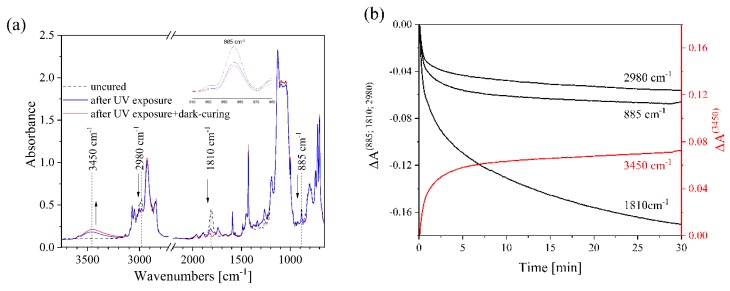
(**a**) FTIR spectra of CEOS before and after 30 min of UV exposure and 24 h of following dark-curing, (inset: zoomed IR range of C–H stretching of epoxy groups in the fingerprint region); (**b**) Evolution of IR absorption of CEOS during UV light exposure: C–H stretching of epoxy groups (885 and 2980 cm^−1^); formation of hydroxyl groups (3450 cm^−1^); decomposition of photoinitiator (1810 cm^−1^).

Figure 9 displays the changes in IR absorption of CEOS occurring upon UV light exposure. Band area at 1810 cm^−1^ decreases during UV irradiation indicating photoinitiator (PI) photodecomposition (Figure 9b). This is followed by formation of radicals and ionic fragments leading to the generation of a strong Brönsted acid in the presence of a hydrogen donor [12,30] (Supplementary Materials Scheme S1). Formed super acid acts as a primary initiator of ring-opening polymerization of the epoxide. This process proceeds rapidly through the oxonium ion and can be seen in Figure 9 by a decrease of the characteristic band at 885 cm^−1^ from epoxy ring vibrations [31,32]. It was supported by a decline in the absorbance at 2980 cm^−1^ associated with C–H stretching in the oxirane ring. Moreover, the band at 3450 cm^−1^ rises as a result of the hydroxyl group formation during the ring-opening. After the exposure was terminated, the crosslinking processes proceeded in the dark as is typical for cationic polymerization [11]. For CEOS, this effect was observed by the absorbance changes displayed by the FTIR spectrum of the film kept for 24 h in the darkness after being UV exposed (Figure 9a: after UV exposure + dark-curing). 

**Figure 10:**
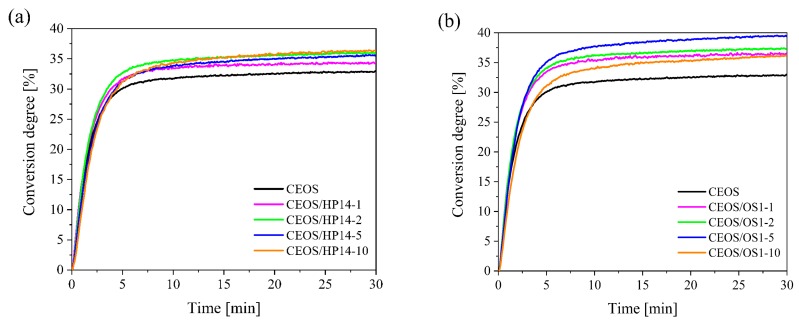
Photopolymerization profiles of neat CEOS and its nanocomposites with different (**a**) HP14 and (**b**) OS1 loadings.

The extent of photopolymerization in neat CEOS and CEOS/BA nanocomposites is shown in Figure 10, as the changes in the conversion degree of epoxy groups (α_ep_) versus exposure time. While the curve slope gives an indication of the polymerization rate, the plateau value identifies the final conversion efficiency. A similar evolution was observed for bands at 2980 cm^−1^ and 3450 cm^−1^ related to a decrease of C–H stretching in oxirane ring and the formation of hydroxyl groups, respectively (Supplementary Materials Figures S9 and S10). 

It was observed that two stages in the cationic photopolymerization take place. The first stage at low-curing times exhibited a fast increase in epoxy group conversion (α_ep_), up to roughly 30%. The second stage, at higher conversion degrees, represents a decelerating CEOS conversion rate that can be explained by the formation of a glassy network. It resulted in a significant drop of mobility of reactive groups due to gelation and vitrification phenomena. Consequently, a large number of epoxy groups remains trapped within the polymeric network with no possibility to reach a neighboring oxirane ring. Therefore, one can observe only a 2% increase in epoxy conversion degree during the last 20 min of UV irradiation. 

**Figure 11:**
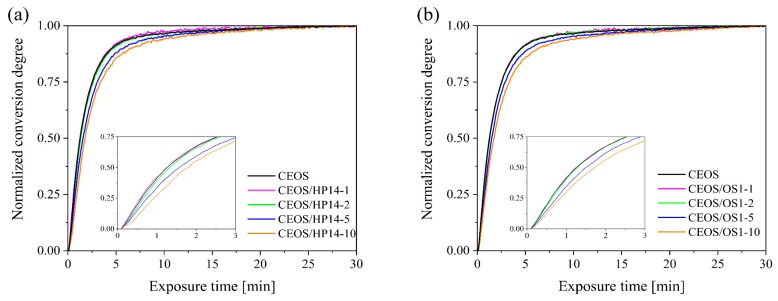
Normalized conversion degree of neat CEOS and its nanocomposites with different (**a**) HP14 and (**b**) OS1 loadings.

For better comparison of kinetics in neat CEOS and nanocomposites, the normalized epoxy conversion curves were calculated (Figure 11). 

As one can see, the induction period of the kinetics extends with particle loading (Figure 11, inset). At the same time, vitrification processes during photocuring appear to be somewhat retarded for the nanocomposites with higher particle content (5–10 wt.%). The inhibition caused by particles during the induction period can be better identified from the first derivative of changes in epoxy band ΔA^885^ (Supplementary Materials Figure S11). However, the possible impact of nanoparticles on the penetration depth of UV light should also be considered as one of the possible factors influencing the photopolymerization kinetic. 

While the first stage of CEOS photopolymerization can be considered an autocatalytic (kinetic controlled), the second stage was controlled by the diffusion occurring in the partially crosslinked polymer at vitrification. High loadings of boehmite particles seemed to influence this process. 

For better understanding of BA’s influence on the network formation of CEOS resin, no additional exposure to the heat or moisture during post-curing was applied in our experiment, thus excluding additional influences after UV light exposure was terminated. 

**Figure 12:**
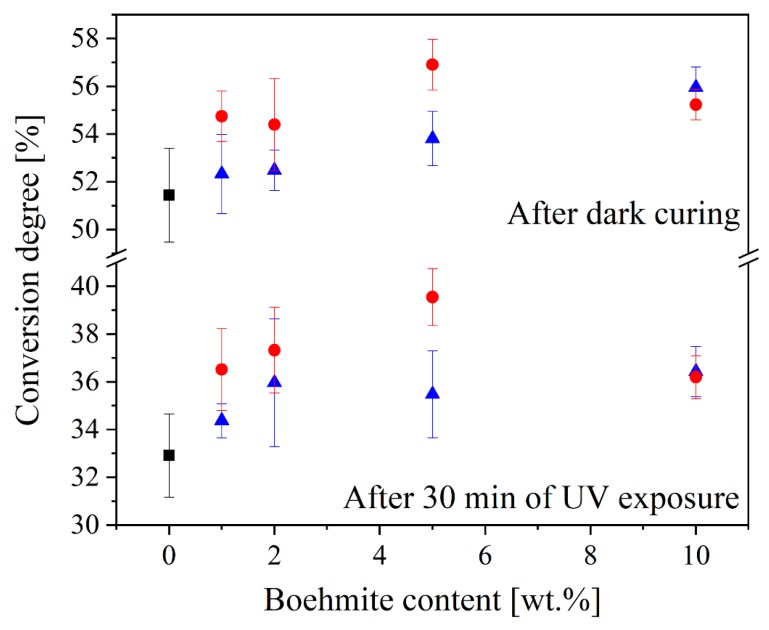
Dependence of conversion degree of CEOS on boehmite content in nanocomposites after 30 min of UV exposure and after subsequent 24 h dark curing period: (■: CEOS; ▲: CEOS/HP14; ●: CEOS/OS1).

Figure 12 depicts the dependence of the final α_ep_ on the BA content detected right after UV irradiation and consequently after the following 24 h of exposed samples resting in the dark. 

It is known that cationic centers of polymerization are not reactive towards each other and therefore have much longer lifetimes in contrast to free-radical polymerization. As a result, cationic polymerization proceeds even after irradiation has been terminated, when the active species are no longer being created. It can be noticed from Figure 12 that the α_ep_ of 33% detected for neat hybrid after 30 min of UV light exposure remarkably increased up to 51% during dark-curing. 

In the case of unmodified boehmite HP14, a statistically significant increase in the nanocomposite’s α_ep_ compared to neat CEOS was observed only at 10 wt.% particle loading. The α_ep_ of 36% and 56% was detected after UV exposure and after dark-curing, respectively. 

At the same time, considering the compositions with organically-modified boehmite, the maximum α_ep_ was detected for CEOS/OS1-5% composition with values of 40% and 57% after irradiation and following post-curing, respectively. These values are by 7% and 6% higher than those found for the neat CEOS. 

As one can see, no reduction in a degree of curing was caused by boehmite embedding, neither after UV exposure stage nor after post-curing in the dark. At the same time, an increase in conversion detected after dark-curing is observed for all studied formulations and is in 17–18% range. This indicates that the differences caused by the two different types of boehmite nanoparticles originate mainly from the processes occurring during the UV light exposure stage. 

### 3.5. Proposed Curing Mechanism

Summarizing the results obtained within RT-IR experiment, we can conclude that the mobility restriction of the active sites of the CEOS network appearing during crosslinking/polymerization limits the final conversion degree of epoxy groups up to roughly only 50%. The incorporation of BA at 5–10 wt.% to CEOS matrix results in a slight decrease in curing rate during the first stage of polymerization, before the gelation occurs. However, surprisingly, the presence of BA has led to an increase in overall α_ep_ of the CEOS network due to the contribution of the processes occurring in the already somewhat vitrified matrix structure. The observed effect excludes previously detected phenomena like a light screening effect of boehmite nanoparticles [33] or a decrease in mobility of the reactive species due to the raised viscosity [5]. These phenomena were shown to induce an opposite effect in other cycloaliphatic epoxy systems where a significant drop of epoxy conversion degree was observed [5,33]. 

Since CEOS films prepared for DSC and TGA analysis as well as T-SEM imaging were cured using different irradiation sources and exposure times due to technical reasons, one cannot directly compare these results to RT-IR curing experiments. Nevertheless, the results from these methods indicate that BA addition leads to a decrease in mobility of cured CEOS network. 

**Scheme 2:**
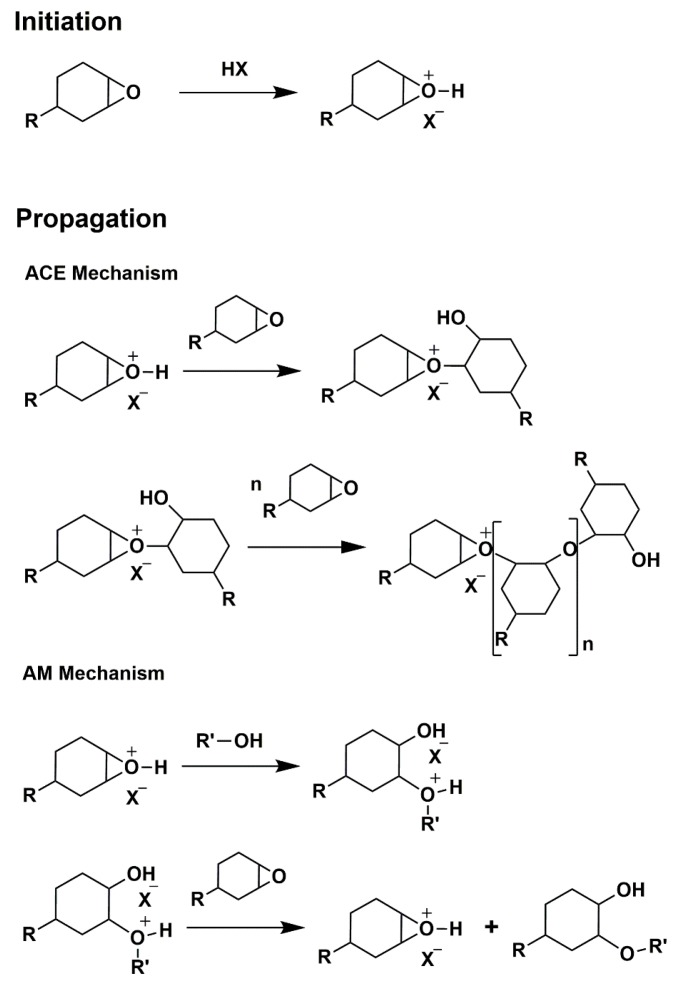
Simplified representation of cationic polymerization of cycloaliphatic epoxy groups: Active Monomer (AM) and Active-Chain End (ACE) mechanisms.

**Scheme 3:**
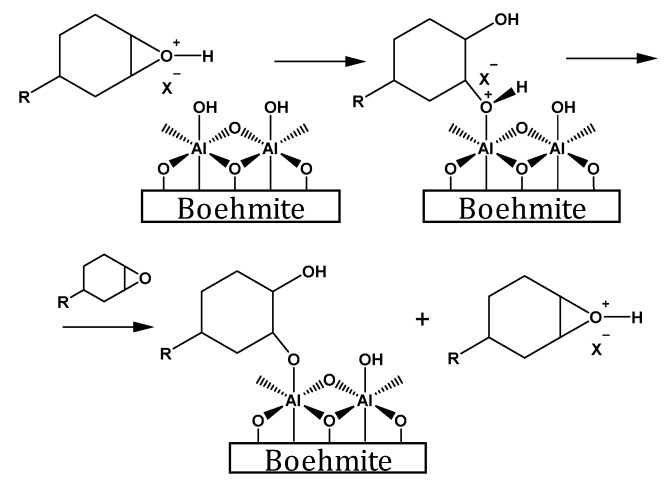
Reaction between oxonium ion and Al–OH groups of boehmite via AM mechanism.

The positive impact of boehmite on the final epoxy conversion that was observed in our investigations can be explained through the presence of hydroxyl groups located on the particle surface as they can be involved in the propagation step of epoxy polymerization. Generally, the propagation can proceed through the subsequent attacks of epoxy groups by oxonium ions as described by active-chain end (ACE) mechanism. However, it can be shifted to active monomer (AM) mechanism through alcohol attack and proton-transfer reactions because of the presence of protonic nucleophiles [34,35,36]. Both mechanisms are displayed in Scheme 2 and described in detail elsewhere [34,37]. The hydroxyl groups carried on the boehmite surface can react with oxirane ring via an AM mechanism (Scheme 3) forming covalent linkages. This fact could explain the significant rise of T_g_ and increased thermal stability of “inorganic” components of nanocomposites (as seen in Figure 5), even when only 1 wt.% of boehmite was present. 

The interaction between Al–OH groups and epoxy has been also previously considered [38,39]. The reaction between exposed hydroxyl groups of boehmite and neutral epoxy group should be also taken into account as another possible reaction mechanism resulting in covalent bonding [39]. 

As was detected from IR spectra and TGA, both boehmites, OS1, and HP14, contain certain amounts of physically absorbed water. This nucleophile is known to influence the cationic polymerization, and therefore, is expected to be another cause boosting the polymerization processes. Water molecule should be considered during cationic ring-opening polymerization to act as a chain-transfer agent as for active-chain end (ACE) so for active monomer (AM) mechanisms (Supplementary Materials Scheme S2), thus, leading to re-initialization [37,40,41]. This was also the case for the previous studies where a small amount of water present in clays caused enhancement in the final epoxy group conversion [42]. 

A higher improvement of different parameters such as epoxy conversion degree and glass transition temperature were observed with use of organo-modified boehmite (OS1) compared to unmodified nanoparticles (HP14). This effect can be explained by improved particle spatial distribution as well as higher dissociation constant of p-toluenesulfonic acid. This modifying agent can in fact interfere with the photoinitiator producing photons, thus, initiating the epoxy groups located at the BA-CEOS interphase. 

## 4. Conclusions

Novel boehmite-embedded organic/inorganic nanocomposites based on cycloaliphatic epoxy oligosiloxane resin were prepared with two different boehmite fillers via UV-induced cationic ring-opening photopolymerization. Material properties such as curing kinetic, epoxy conversion degree, particles distribution, glass transition and thermal stability were investigated with regard to various loading amounts of boehmite nanoparticles. 

First, an increase of glass transition temperature was found for all nanocomposites indicating reduced mobility of chain segments in cured hybrid network. The strong reinforcement effect was already detected at 1 wt.% boehmite loading. Second, improved thermal stability indicated attractive particle-resin interactions. 

The particles dispersion can be described as a homogeneously distributed network of cluster-like agglomerates of boehmite. The size of detected agglomerates was generally bigger for unmodified boehmite, due to the insufficient polymer–particle interaction. 

The vitrification of the CEOS network had somewhat retarded with the addition of BA; the overall epoxy conversion was increased for nanocomposites with 5–10 wt.% of boehmite. These results are contradictory to the previous studies on the cycloaliphatic epoxy/BA systems where the decreased monomer mobility due to raised viscosity and/or light screening effect of the boehmite particles caused the hindering of cross-linking processes in the polymer. However, in our study, the vitrification appears to be the main limitation factor preventing reaching a fully polymerized structure. 

The elevated α_ep_ of CEOS/BA nanocomposites compared to the neat resin film can be explained by several reasons. First, hydroxyl groups present at boehmite’s surface might react with oxonium ion via an active monomer (AM) polymerization mechanism. The second reason is the contribution of chain-transfer reactions initiated by nucleophilic water introduced into the system with boehmite nanoparticles. Third is the additional proton production induced by the presence of p-toluenesulfonic acid as a surface modifier. 

The impact of particle surface on nanocomposite behavior is evident as it defines the nature of polymer–particle interactions. With organo-modified boehmite nanoparticles (OS1), we achieved the nanocomposites with better spatial particle distribution, higher final epoxy group conversion, as well as higher T_g_ values compared to nanocomposite with unmodified particles (HP14). On the one hand, larger polymer–particle interphase resulted from the better particle–polymer compatibility and particle distribution. On the other hand, positive contribution to the initiation processes can be explained by the presence of p-toluenesulfonic acid as well. 

## Figures and Tables

**Table 1:** Physio-chemical properties of employed boehmite (BA) nanoparticles [21].

	DISPERAL^®^ HP14	DISPERAL^®^ OS1
Mean crystallite size (120) [nm]	14	10
Surface area ^1^ (m^2^/g)	160	240
Loose bulk density (g/cm^3^)	400–600	400–600
Surface treatment	-	p-toluenesulfonic acid

^1^ Calculated according to Brunauer–Emmett–Teller (BET) theory, activation at 550 °C for 3 h.

**Table 2:** Thermal properties of CEOS and CEOS/BA nanocomposites during second heating run: glass transition at half height (T_g_) and heat capacity (C_p_).

	T_g_ (°C)	ΔC_p_ (J/g/°C)
CEOS	30 ± 2	0.23 ± 0.01
CEOS/HP14-1%	52 ± 3	0.20 ± 0.02
CEOS/HP14-2%	54 ± 2	0.16 ± 0.04
CEOS/HP14-5%	48 ± 2	0.16 ± 0.02
CEOS/HP14-10%	45 ± 3	0.15 ± 0.02
CEOS/OS1-1%	64 ± 5	0.10 ± 0.06
CEOS/OS1-2%	73 ± 4	0.05 ± 0.05
CEOS/OS1-5%	71 ± 3	0.07 ± 0.02
CEOS/OS1-10%	65 ± 4	0.08 ± 0.01

## Supplementary Materials

The following are available online at https://www.mdpi.com/1996-1944/12/9/1513/s1, Figure S1: Structures of (a) unmodified and (b) p-toluenesulfonic acid modified boehmite. Figure S2: ^13^C NMR spectra of CEOS synthesized at different ECTS:DPSD molar ratios. Figure S3: FTIR spectra of boehmite powders: unmodified, HP14 and surface modified with p-toluenesulfuric acid, OS1. Figure S4: DSC curves at second heating of CEOS without and with different boehmite loadings: (a) HP14 and (b) OS1. Figure S5: Determination of the glass transition temperature for CEOS hybrid. Figure S6: Determination of the glass transition temperature for CEOS/HP14 compositions. Figure S7: Determination of the glass transition temperature for CEOS/HP14 compositions. Figure S8: (a) TG and (b) DTG curves of HP14 and OS1 boehmite powders. Figure S9: Decrease of C–H stretching of oxirane ring during UV irradiation for CEOS with (a) HP14 and (b) OS1 boehmite nanoparticles. Figure S10: Formation of hydroxyl groups yielded from oxirane ring opening for CEOS with (a) HP14 and (b) OS1 boehmite nanoparticles. Figure S11: First derivative of curing kinetics of neat CEOS and its nanocomposites with different (a) HP14 and (b) OS1 loadings. Table S1. Characteristic IR bands of CEOS and BA in middle infrared region. Scheme S1: (a) Chemical structure and (b) simplified scheme of photodecomposition of arylsulfonium hexafluorophosphate salt. Scheme S2: Reaction of epoxy with water under acidic conditions.

## References

[1] Müller K., Bugnicourt E., Latorre M., Jorda M., Echegoyen Sanz Y., Lagaron J.M., Miesbauer O., Bianchin A., Hankin S., Bölz U. (2017). Review on the Processing and Properties of Polymer Nanocomposites and Nanocoatings and Their Applications in the Packaging, Automotive and Solar Energy Fields. Nanomaterials.

[2] Silvestre J., Silvestre N., De Brito J. (2016). Polymer nanocomposites for structural applications: Recent trends and new perspectives. Mech. Adv. Mater. Struct..

[3] Esposito C.C., Annalisa C., Mariaenrica F. (2013). Measurements of size distribution nanoparticles in ultraviolet-curable methacrylate-based boehmite nanocomposites. J. Appl. Polym. Sci..

[4] Hull T.R., Witkowski A., Hollingbery L. (2011). Fire retardant action of mineral fillers. Degrad. Stab..

[5] Sangermano M., Deorsola F.A., Fabiani D., Montanari G., Rizza G. (2009). Epoxy-boehmite nanocomposites as new insulating materials. J. Appl. Sci..

[6] Ghasem Zadeh Khorasani M., Silbernagl D., Szymoniak P., Hodoroaba V.-D., Sturm H. (2019). The effect of boehmite (AlOOH) on nanomechanical and thermomechanical properties correlated to crosslinking density of epoxy in epoxy/boehmite nanocomposites. Polymer.

[7] Karger-Kocsis J., Lendvai L. (2018). Polymer/boehmite nanocomposites: A review. J. Appl. Polym. Sci..

[8] Shefer K.I., Cherepanova S.V., Moroz É.M., Gerasimov E.Y., Tsybulya S.V. (2010). Features of the real structure of pseudoboehmites: Violations of the structure and layer packing caused by crystallization water. J. Struct. Chem..

[9] Serra A., Ramis X., Fernández-Francos X. (2016). Epoxy Sol-Gel Hybrid Thermosets. Coatings.

[10] Lebeau B., Sanchez C. (1999). Sol-gel derived hybrid inorganic-organic nanocomposites for optics. Curr. Opin. Solid State Mater. Sci..

[11] Decker C., Moussa K. (1990). Kinetic investigation of photopolymerizations induced by laser beams. Macromol. Chem..

[12] Decker C., Viet T.N.T., Thi H.P. (2001). Photoinitiated cationic polymerization of epoxides. Polym. Int..

[13] Golaz B., Michaud V., Leterrier Y., Månson J.-A. (2012). UV intensity, temperature and dark-curing effects in cationic photo-polymerization of a cycloaliphatic epoxy resin. Polymer.

[14] Sangermano M., Razza N., Crivello J.V. (2014). Cationic UV-Curing: Technology and Applications. Macromol. Mater. Eng..

[15] Zhu Q., Liang L., Du X., Xiao F., Guo Y., Shi J., Wu K., Lu M. (2018). Fabrication of High-Performance Cationic UV Curable Cycloaliphatic Epoxy/Silicone Hybrid Coatings. Macromol. Mater. Eng..

[16] Teramoto N., Kogure H., Kimura Y., Shibata M. (2004). Thermal properties and biodegradability of the copolymers of l-lactide, ε-caprolactone, and ethylene glycol oligomer with maleate units and their crosslinked products. Polymer.

[17] Ho J.K., Byeong-Soo B. (2008). Synthesis and characterization of photopatternable epoxy hybrid materials for the fabrication of thick and thermally stable microstructures with a high aspect ratio. J. Appl. Polym. Sci..

[18] Jin J., Lee J.J., Bae B.-S., Park S.J., Yoo S., Jung K. (2012). Silica nanoparticle-embedded sol–gel organic/inorganic hybrid nanocomposite for transparent OLED encapsulation. Org. Electron..

[19] Topolniak I., Chapel A., Gaume J., Bussiere P.-O., Chadeyron G., Gardette J.-L., Therias S., Chadeyron G. (2017). Applications of polymer nanocomposites as encapsulants for solar cells and LEDs: Impact of photodegradation on barrier and optical properties. Degrad. Stab..

[20] Xalter R., Halbach T.S., Mülhaupt R. (2006). New Polyolefin Nanocomposites and Catalyst Supports Based on Organophilic Boehmites. Macromol. Symp..

[21] www.products.sasol.com. https://products.sasol.com/pic/products/home/grades/ZA/5disperal-and-dispal/index.html.

[22] Hodoroaba V.-D., Rades S., Unger W.E.S. (2014). Inspection of morphology and elemental imaging of single nanoparticles by high-resolution SEM/EDX in transmission mode. Surf. Interface Anal..

[23] Stuart B.H. (2004). Infrared Spectroscopy: Fundamentals and Applications.

[24] Jung K., Bae J.-Y., Park S.J., Yoo S., Bae B.-S. (2011). High performance organic-inorganic hybrid barrier coating for encapsulation of OLEDs. J. Mater. Chem..

[25] Tipson R.S. (1952). Infrared Absorption Spectra of p-Toluenesulfonic Acid and of Some of Its Esters. J. Am. Chem. Soc..

[26] Goertzen W., Kessler M., Kessler M. (2007). Thermal and mechanical evaluation of cyanate ester composites with low-temperature processability. Compos. Part A Appl. Sci. Manuf..

[27] Mutlur S., Kessler M.R. (2004). Thermal Analysis of Composites Using DSC. Advanced Topics in Characterization of Composites.

[28] Bokhimi X., Toledo-Antonio J., Guzmán-Castillo M., Hernández-Beltrán F. (2001). Relationship between Crystallite Size and Bond Lengths in Boehmite. J. Solid State Chem..

[29] Fankhänel J., Silbernagl D., Khorasani M.G.Z., Daum B., Kempe A., Sturm H., Rolfes R. (2016). Mechanical Properties of Boehmite Evaluated by Atomic Force Microscopy Experiments and Molecular Dynamic Finite Element Simulations. J. Nanomater..

[30] Odian G. (2004). Ionic Chain Polymerization. Principles of Polymerization.

[31] Jabbour J., Calas S., Gatti S., Kribich R., Myara M., Pille G., Etienne P., Moreau Y. (2008). Characterization by IR spectroscopy of an hybrid sol–gel material used for photonic devices fabrication. J. Non-Crystalline Solids.

[32] Gao N., Liu W., Yan Z., Wang Z. (2013). Synthesis and properties of transparent cycloaliphatic epoxy–silicone resins for opto-electronic devices packaging. Opt. Mater..

[33] Corcione C.E., Frigione M., Maffezzoli A., Malucelli G. (2008). Photo – DSC and real time – FT-IR kinetic study of a UV curable epoxy resin containing o-Boehmites. Eur. J..

[34] Park S.-J., Heo G.-Y., Suh D.-H. (2003). Thermal properties and fracture toughness of epoxy resins cured by phosphonium and pyrazinium salts as latent cationic initiators. J. Sci. Part A Chem..

[35] Penczek S., Kubisa P., Szymanski R. (1986). Activated monomer propagation in cationic polymerizations. Makromol. Chemie Macromol. Symp..

[36] Li Y.-S., Li M.-S., Chang F.-C., Li Y., Chang F. (1999). Kinetics and curing mechanism of epoxy and boron trifluoride monoethyl amine complex system. J. Sci. Part A Chem..

[37] Tokar R., Kubisa P., Penczek S., Dworak A. (1994). Cationic polymerization of glycidol: coexistence of the activated monomer and active chain end mechanism. Macromolecules.

[38] Akatsuka M., Takezawa Y., Amagi S. (2001). Influences of inorganic fillers on curing reactions of epoxy resins initiated with a boron trifluoride amine complex. Polymer.

[39] Rajabi L., Marzban M., Derakhshan A.A. (2014). Epoxy/alumoxane and epoxy/boehmite nanocomposites: Cure behavior, thermal stability, hardness and fracture surface morphology. Iran. J..

[40] Kubisa P., Penczek S. (1999). Cationic activated monomer polymerization of heterocyclic monomers. Prog. Sci..

[41] Choi G.-M., Jin J., Shin D., Kim Y.H., Ko J.-H., Im H.-G., Jang J., Jang D., Bae B.-S. (2017). Flexible Hard Coating: Glass-Like Wear Resistant, Yet Plastic-Like Compliant, Transparent Protective Coating for Foldable Displays. Adv. Mater..

[42] Bongiovanni R., Turcato E.A., Di Gianni A., Ronchetti S. (2008). Epoxy coatings containing clays and organoclays: Effect of the filler and its water content on the UV-curing process. Prog. Org. Coat..

