# Supplementary material for: Boehmite Nanofillers in Epoxy Oligosiloxane Resins: Influencing the Curing Process by Complex Physical and Chemical Interactions
Source: Materials (Basel). 2019 May 9;12(9):1513. doi: 10.3390/ma12091513 (PMC6540208; doi:10.3390/ma12091513)
Supplement: Supplementary file 1 [file materials-12-01513-s001.pdf]

materials

Supplementary Materials

Boehmite Nanofillers in Epoxy Oligosiloxane Resins: Influencing the Curing Process by Complex Physical and Chemical Interactions

Ievgeniia Topolniak [1,*], Vasile-Dan Hodoroaba [1], Dietmar Pfeifer [1], Ulrike Braun [1] and Heinz Sturm [1,2,*]

[1] Federal Institute for Material Research and Testing (BAM), 12205 Berlin, Germany; Dan.Hodoroaba@bam.de (V.-D.H.); Dietmar.Pfeifer-Berlin@t-online.de (D.P.); ulrike.braun@bam.de (U.B.)
[2] Institute Machine Tools and Factory Management, Technical University Berlin, 10587 Berlin, Germany
* Correspondence: ievgeniia.topolniak@gmail.com (I.T.); heinz.sturm@bam.de (H.S.)

1. Materials and film curing

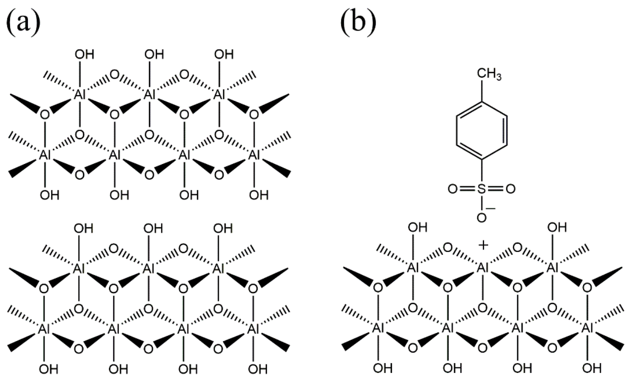

Figure S1. Structures of (a) unmodified and (b) p-toluenesulfonic acid modified Boehmite (adapted from [1]).

2. Characterization

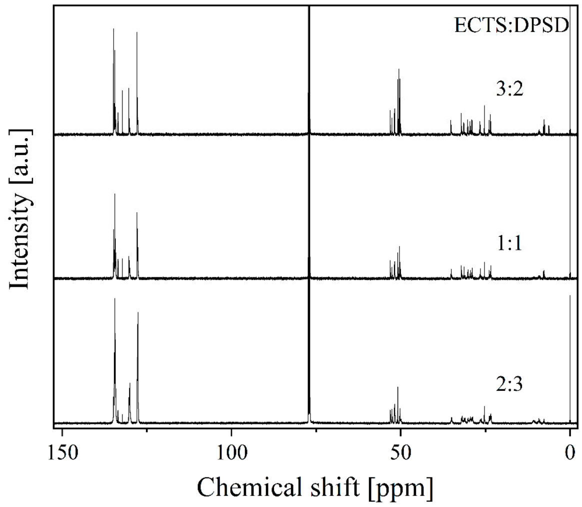

Figure S2. ^{13}C NMR spectra of CEOS synthesized at different ECTS:DPSD molar ratios**.**

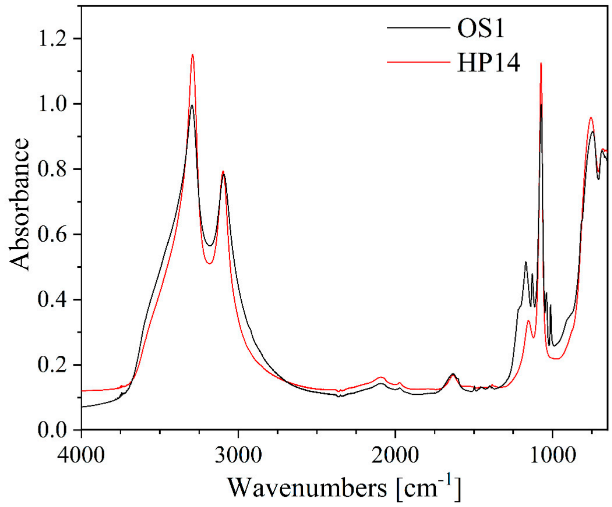

Figure S3. FTIR spectra of Boehmite powders: unmodified, HP14 and surface modified with p-toluenesulfuric acid, OS1.

The characteristic IR bands of CEOs and Boehmite are summarized in Table S1.

Table 1. Characteristic IR bands of CEOS and BA in middle infrared region [2] [3] [4] [5].

**Wavenumber [cm^{-1}]**	**Assignment**
750, 680	OH out-of-plane bending (BA)
885	C-O-C stretching of oxirane ring
1040-1090	Si-O-Si asymmetric stretching
1430, 1110	Si-Ph
1064	OH in-plane bending (BA)

1120-1130	Si-Ph
1810	Photoinitiator
2840	Si-OMe stretching
2925	C-H asymmetric stretching (CH_2, aliphatic)
2980	C-H asymmetric stretching in oxirane ring
3070,3050	C-H stretching of phenyl ring
3290, 3100	OH stretching (BA)
3450	OH stretching

3. Determination of Glass transition temperature (T_g)

Glass transition of neat CEOS and different nanocomposite formulation was evaluated using DSC (Figure S4). T_g was detected as the temperature at half-height of change in heat capacity. The scaled heat flow signal with fitting example of all studied compositions are given in Figure S5 -Figure S7.

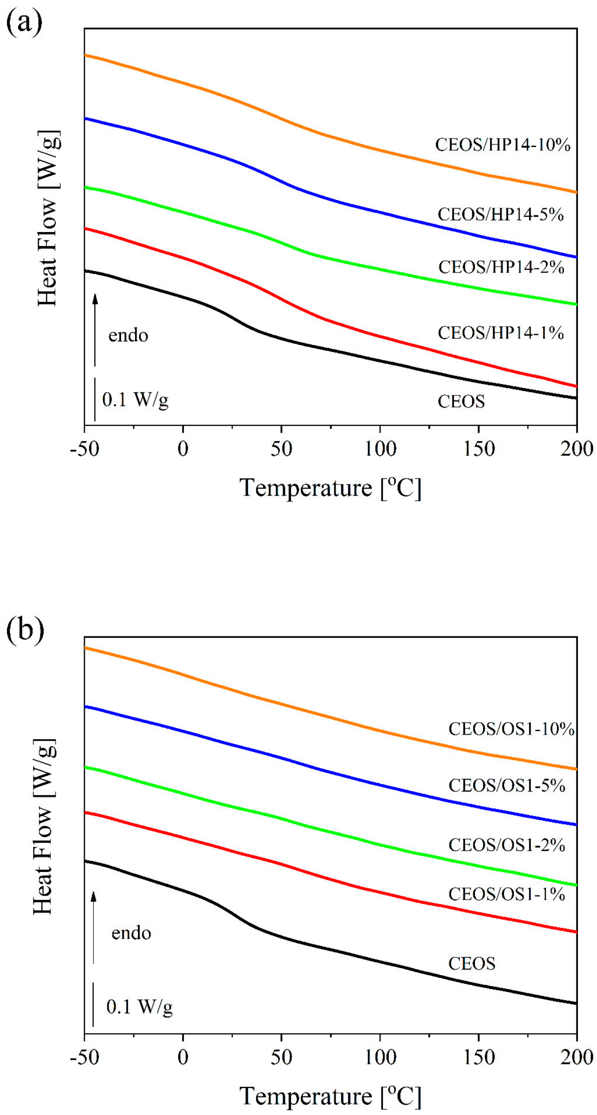

Figure S4. DSC curves at second heating of CEOS without and with different Boehmite loadings: (a) HP14 and (b) OS1.

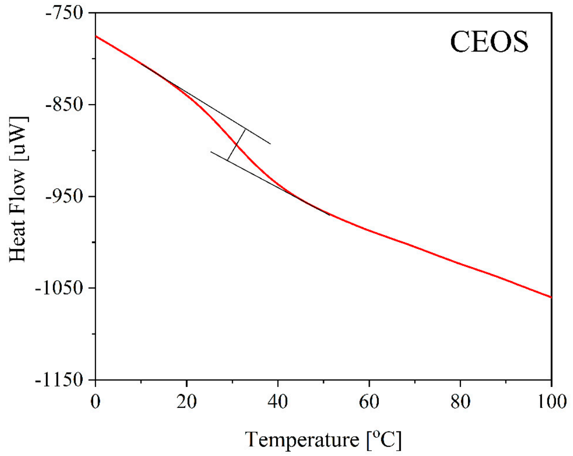

Figure S5. Determination of the glass transition temperature for CEOS hybrid.

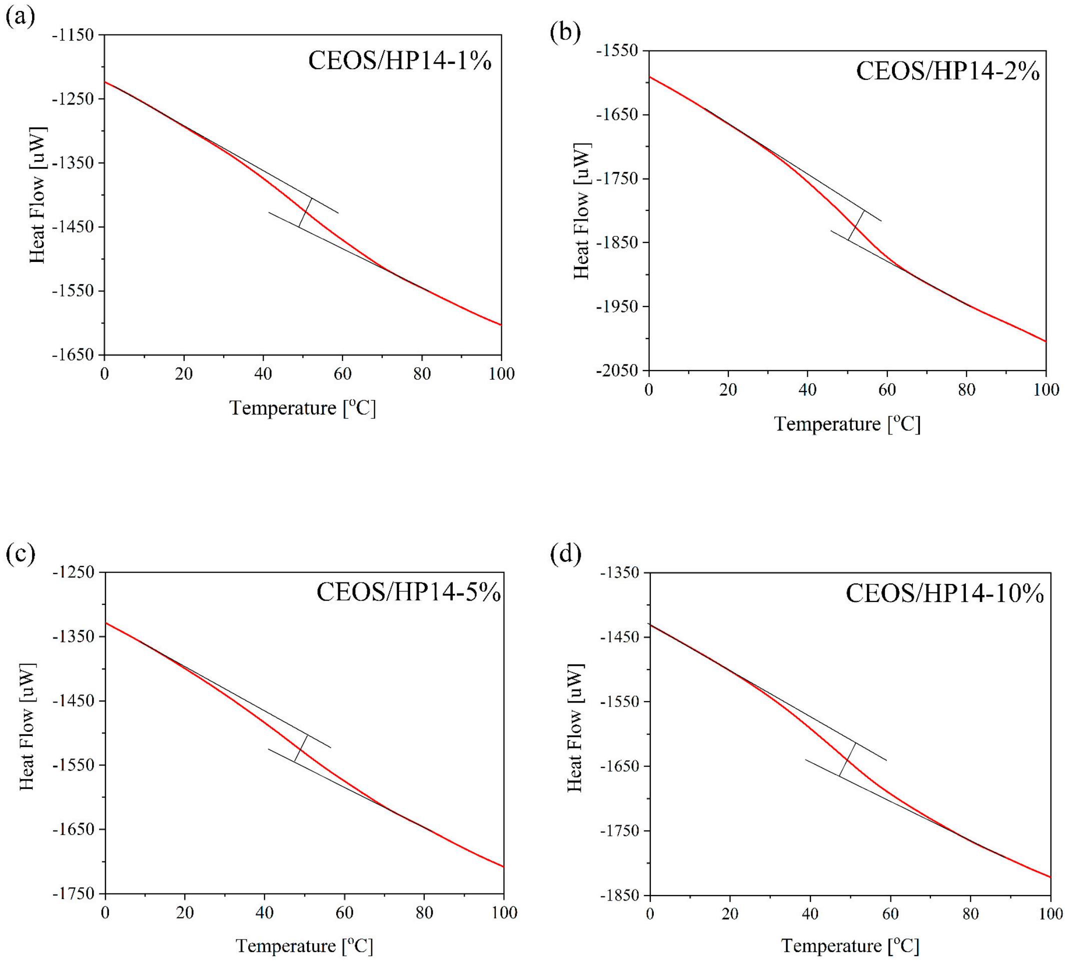

Figure S6. Determination of the glass transition temperature for CEOS/HP14 with different Boehmite loadings: (a) 1%; (b) 2%; (c) 5%; (d) 10%.

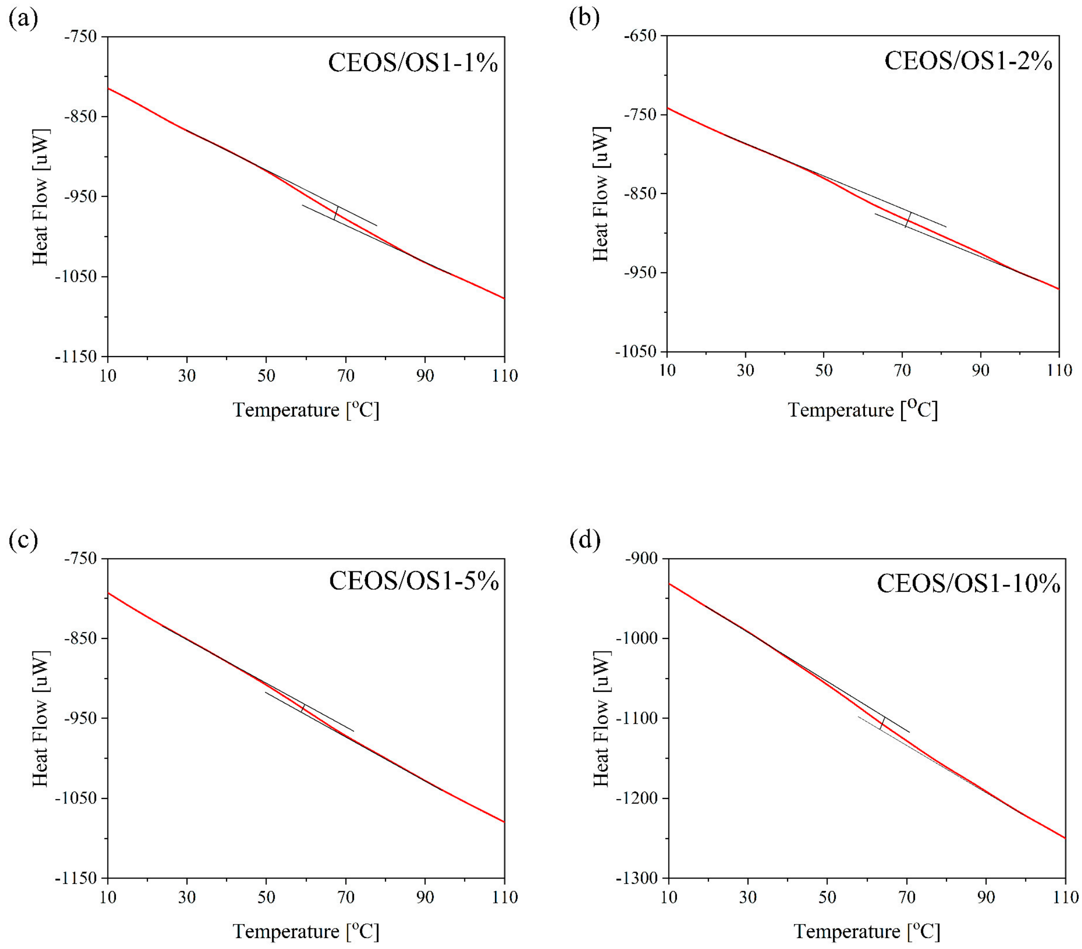

Figure S7. Determination of the glass transition temperature for CEOS/OS1with different Boehmite loadings: (a) 1%; (b) 2%; (c) 5%; (d) 10%.

4. Thermal gravimetric analysis

TGA of Boehmite nanoparticles was carried out in order to determine the present degradation steps in used fillers (Figure S8). Two degradation steps in unmodified Boehmite (HP14) are detectable. The first is observed at temperatures below 100 °C and can be associated with physically absorbed water. The second step, appearing as a main weight loss, take place between 300 °C and 470 °C with the DTG maximum rate at 445 °C. It represents the dihydroxylation of Boehmite leading to formation of Al_2O_3 and H_2O [6].

Four degradation stages can be noticed for organo-modified Boehmite OS1. The first step appears between 30 and 150 °C with the DTG curve maximum at 65 °C. The second step begins at around 300 °C with DTG peak at 445 °C. The third degradation step exhibits shallow DTG peak at 470 °C and partially overlaps with the second. The fourth step begins at 550 °C. The first and second degradation steps is believed to correspond to the same processes as in the unmodified Boehmite, i.e. physical water desorption and dihydroxylation processes. It was suggested that the presence of PTSA in OS1 increases water absorption/desorption rate compared to HP14 what results in a decrease of temperature at which the first two degradation steps appear [7]. In our case higher content of

physically absorbed water for OS1 is detected from weight loss at the first step. The third degradation step, present only in o-Boehmite, is attributed to the decomposition of surface modifier, resulting in sulfates formation [7]. The last fourth step represents decomposition of aluminum sulfate formed in PTSA degradation. The overall weight loss during heating up to 750 °C of Boehmite powders were 27% and 24% for HP14 and OS1, respectively.

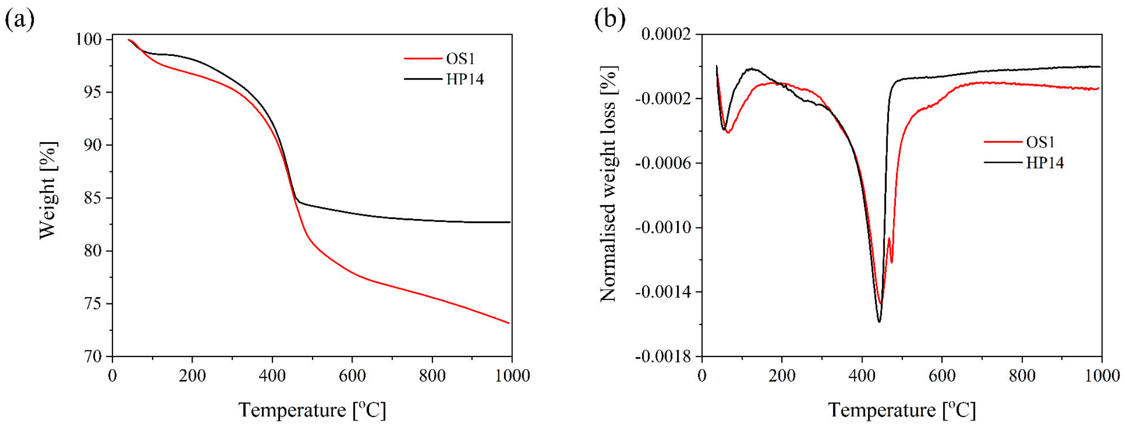

Figure S8. (a) TG and (b) DTG curves of HP14 and OS1 Boehmite powders.

5. Photoinitiator structure and decomposition

The chemical formula of used photoinitiator and simplified representation of photolysis are given below:

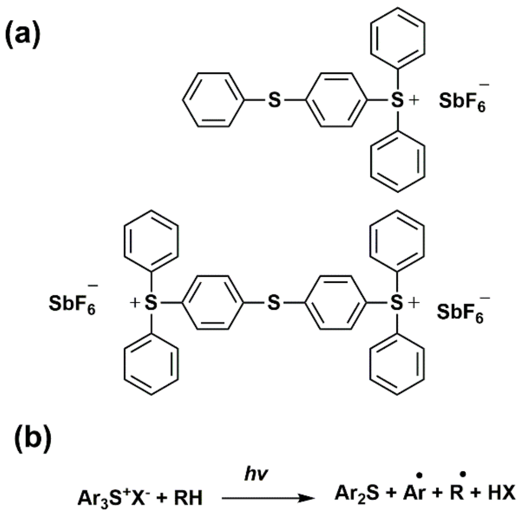

Scheme S1. (a) Chemical structure and (b) simplified scheme of photodecomposition of arylsulfonium hexafluorophosphate salt.

6. Real-Time Infrared spectroscopy: curing kinetics

The evolutions of band absorptions associated with the decrease of C-H stretching in oxirane ring and the formation of hydroxyl groups located at 2980 cm^{-1} and
3450 cm^{-1}, respectively, are given in Figure S9 - Figure S10.

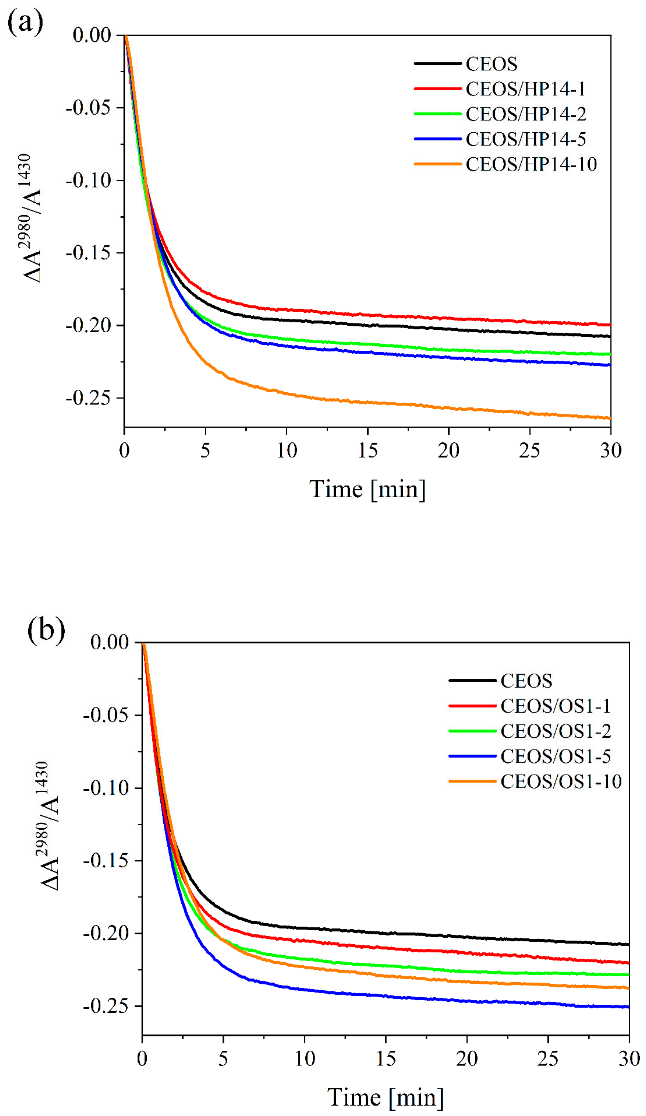

Figure S9. Decrease of C-H stretching of oxirane ring during UV irradiation for CEOS with (a) HP14 and (b) OS1 Boehmite nanoparticles.

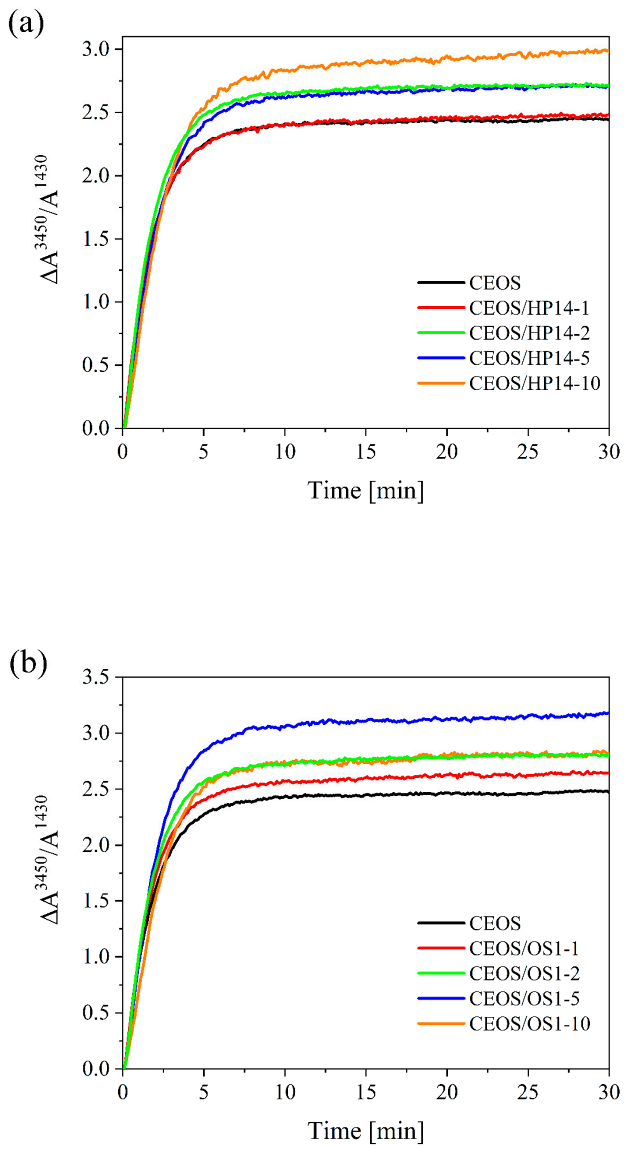

Figure S10. Formation of hydroxyl groups yielded from oxirane ring opening for CEOS with (a) HP14 and (b) OS1 Boehmite nanoparticles.

First derivative of peak area changes is given in Figure S11. The deceleration at the beginning of UV irradiation is observed for CEOS/BA nanocomposites, which becomes more pronounced with an increase of Boehmite concentration.

Figure S11. First derivative of curing kinetics of neat CEOS and its nanocomposites with different (a) HP14 and (b) OS1 loadings.

7. The effect of moisture on epoxy conversion

It is believed that water has no significant influence on the photodecomposition of triarylsulfonium salts. Water might play a solvating role, but it is assumed that it does not impact the number of active sites. Two competitive mechanisms involving water molecules as a chain-transfer agent should be considered during cationic ring-opening polymerization, so-called active-chain end

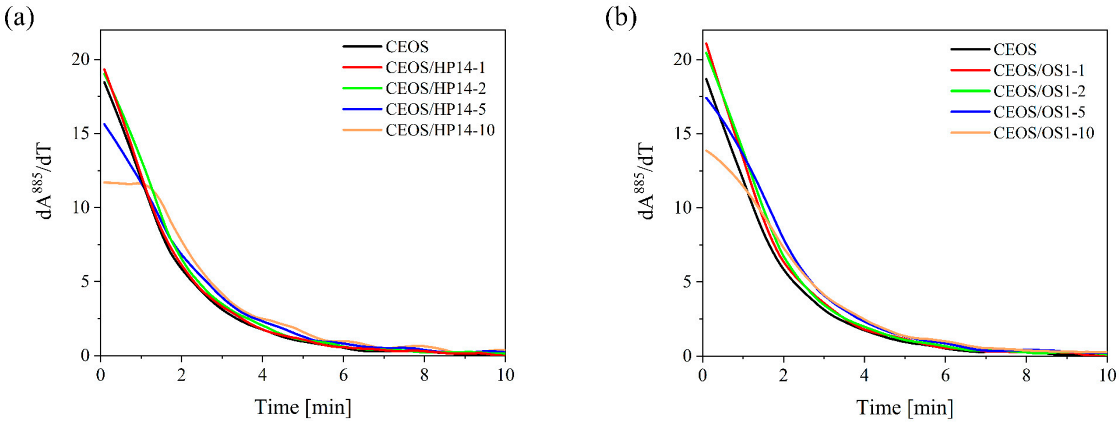

(ACE) and active monomer (AM) mechanism [8] [9] [10]. The mechanisms of cationic ring-opening involving water are illustrated in the following Scheme.S 2.

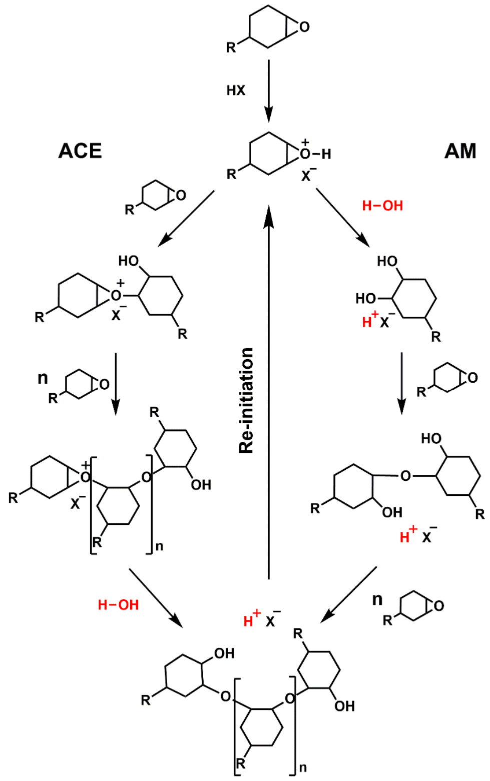

Scheme.S2. Reaction of epoxy with water under acidic conditions (adapted from [10]).

Absence of a chain transfer agent (in our case water molecules) leads to the ACE reaction mechanism resulting in the subsequent isolation of active sites and therefore gradual decrease of polymerization rate. This phenomenon is observed for CEOS photocuring. The addition of water, for instance, during moisture post-annealing, into reaction-limited polymer matrix results in reaction between H_2O molecule and tertiary oxonium ion yielding a hydroxyl end group and an acidic proton that reinitiates a neutral epoxide. On the other side, an ample amount of H_2O leads to polymerization followed by AM mechanism leading to significant increase of polymerization events as it was found for moisture post-treatment of cycloaliphatic epoxy [10].

1. Xalter, R.; Halbach, T.S.; Mülhaupt, R. New Polyolefin Nanocomposites and Catalyst Supports Based on Organophilic Boehmites. *Macromolecular Symposia* **2006**, *236*, 145-150, doi:doi:10.1002/masy.200690048.
2. Stuart, B. *Infrared spectroscopy: fundamentals and applications*; Chichester, West Sussex, England ; Hoboken, NJ : J. Wiley: 2004; doi:10.1002/0470011149.
3. Jabbour, J.; Calas, S.; Gatti, S.; Kribich, R.K.; Myara, M.; Pille, G.; Etienne, P.; Moreau, Y. Characterization by IR spectroscopy of an hybrid sol–gel material used for photonic devices fabrication. *Journal of Non-Crystalline Solids* **2008**, *354*, 651-658, doi:10.1016/j.jnoncrysol.2007.07.091.
4. Houbertz, R.; Domann, G.; Cronauer, C.; Schmitt, A.; Martin, H.; Park, J.U.; Fröhlich, L.; Buestrich, R.; Popall, M.; Streppel, U., et al. Inorganic–organic hybrid materials for application in optical devices. *Thin Solid Films* **2003**, *442*, 194-200, doi:10.1016/S0040-6090(03)00982-9.
5. Koichumanova, K.; Sai Sankar Gupta, K.B.; Lefferts, L.; Mojet, B.L.; Seshan, K. An in situ ATR-IR spectroscopy study of aluminas under aqueous phase reforming conditions. *Physical chemistry chemical physics : PCCP* **2015**, *17*, 23795-23804, doi:10.1039/c5cp02168e.
6. Yang, W.P.; Shyu, S.S.; Lee, E.-S.; Chao, A.-C. Effects of PVA content and calcination temperature on the properties of PVA/boehmite composite film. *Materials Chemistry and Physics* **1996**, *45*, 108-113, doi:10.1016/0254-0584(96)80086-1.
7. Esposito Corcione, C.; Frigione, M.; Maffezzoli, A.; Malucelli, G. Photo – DSC and real time – FT-IR kinetic study of a UV curable epoxy resin containing o-Boehmites. *European Polymer Journal* **2008**, *44*, 2010-2023, doi:10.1016/j.eurpolymj.2008.04.030.
8. Tokar, R.; Kubisa, P.; Penczek, S.; Dworak, A. Cationic polymerization of glycidol: coexistence of the activated monomer and active chain end mechanism. *Macromolecules* **1994**, *27*, 320-322, doi:10.1021/ma00080a002.
9. Kubisa, P.; Penczek, S. Cationic activated monomer polymerization of heterocyclic monomers. *Progress in Polymer Science* **1999**, *24*, 1409-1437, doi:10.1016/S0079-6700(99)00028-3.
10. Choi, G.-M.; Jin, J.; Shin, D.; Kim, Y.H.; Ko, J.-H.; Im, H.-G.; Jang, J.; Jang, D.; Bae, B.-S. Flexible Hard Coating: Glass-Like Wear Resistant, Yet Plastic-Like Compliant, Transparent Protective Coating for Foldable Displays. *Advanced Materials* **2017**, *29*, 1700205, doi:doi:10.1002/adma.201700205.
